# Supplementary material for: A neuronal prospect theory model in the brain reward circuitry
Source: Nat Commun. 2022 Oct 4;13:5855. doi: 10.1038/s41467-022-33579-0 (PMC9532451; doi:10.1038/s41467-022-33579-0)
Supplement: Supplementary file 1 — Supplementary Information [file 41467_2022_33579_MOESM1_ESM.pdf]

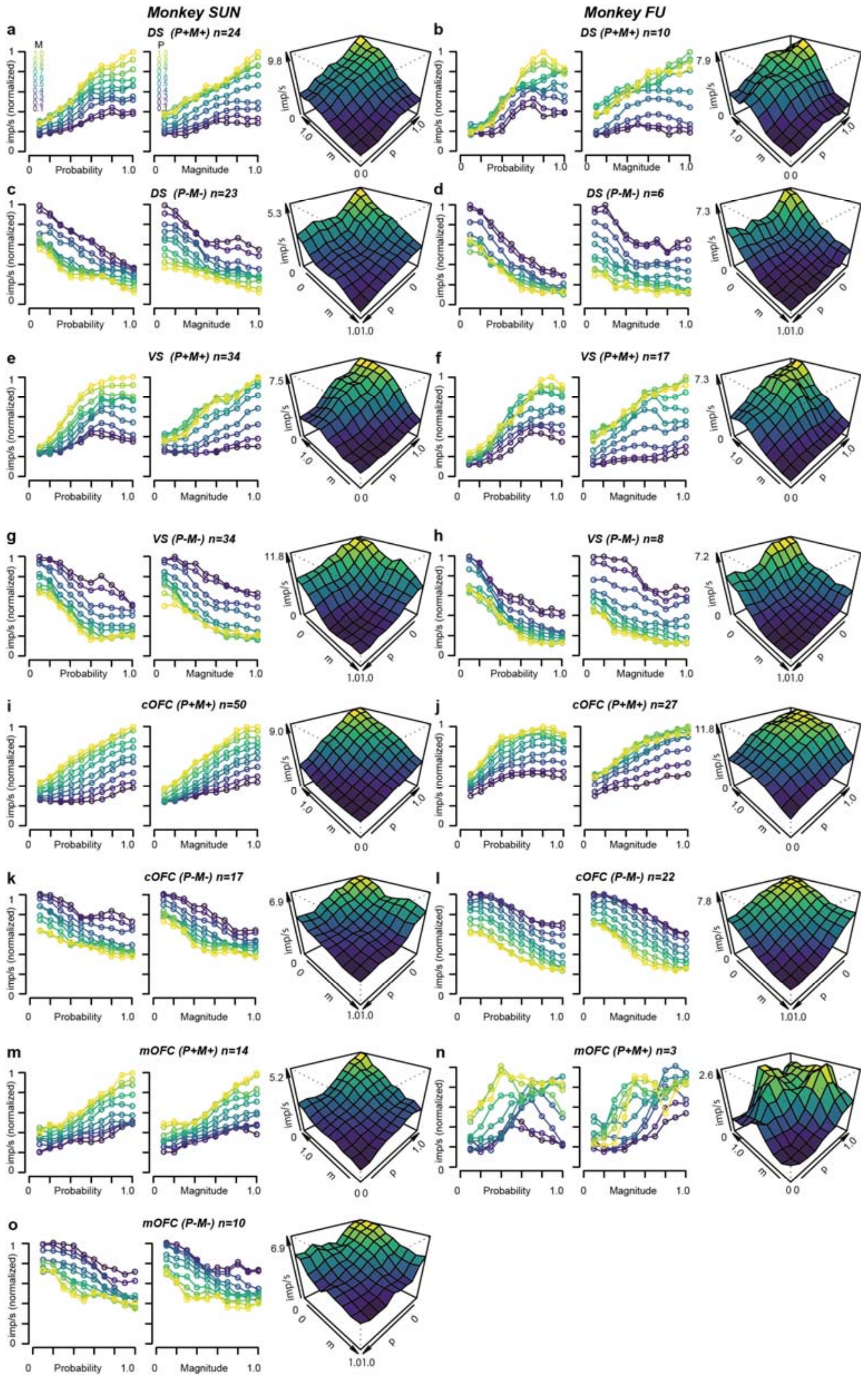
Monkey SUN
Monkey FU
a
DS (P+M+) n=24
b
DS (P+M+) n=10
c
DS (P-M-) n=23
d
DS (P-M-) n=6
e
VS (P+M+) n=34
f
VS (P+M+) n=17
g
VS (P-M-) n=34
h
VS (P-M-) n=8
i
cOFC (P+M+) n=50
j
cOFC (P+M+) n=27
k
cOFC (P-M-) n=17
l
cOFC (P-M-) n=22
m
mOFC (P+M+) n=14
n
mOFC (P+M+) n=3
o
mOFC (P-M-) n=10
imp/s (normalized)
Probability
Magnitude
imp/s
M
P
m
p

Supplementary Figure 1. Population activities encoding probability and magnitude of rewards in the four brain regions

a Population activities of the *P+M+* type recorded in the DS in monkey SUN for 10 levels of probability and magnitude of rewards. P and M indicate the probability and magnitude of rewards, respectively. Firing rates were normalized by the maximum firing rates among the combination of 100 lotteries. A curvature plot of population activities for the 100 lotteries is shown on the right. Average smoothing was made between neighboring pixels; n indicates the number of activities detected among the four analysis epochs. **b** similar to **a** but for monkey FU. **c-d** similar to **a-b** but for *P-M-* type. **e-h** similar to **a-d** but recorded in the DS**.** **i-l** similar to **a-d** but recorded in the cOFC. **m-o** similar to **a-c** but recorded in the mOFC. No neurons for the P-M- type were recorded in the mOFC in monkey FU.
