# Supplementary material for: A neuronal prospect theory model in the brain reward circuitry
Source: Nat Commun. 2022 Oct 4;13:5855. doi: 10.1038/s41467-022-33579-0 (PMC9532451; doi:10.1038/s41467-022-33579-0)
Supplement: Supplementary file 3 — Description of Additional Supplementary Files [file 41467_2022_33579_MOESM3_ESM.pdf]

Description of Additional Supplementary Files

We prepared R code for two important analyses in our manuscript. Neural clustering shown in Figure 5 and simulation results in Figure 6.

File name: Supplementary Data 1

Description: List of estimated parameters of the economic model in each activity of neurons. The scatter plots of these parameters are shown in Fig. 5a. The data was used to make neural cluster as above.

File name: Supplementary Code 1

Description: In Figure 5, Supplementary_Code1.r was used to make neural clusters using R software. In the code, Supplementary_Data.csv was loaded and analyzed. Please read the file in R and run the code.

File name: Supplementary Code 2

Description: In Figure 6, Supplementary_Code2.r was used to perform a simulation in R software. Please load the file in R using source() function. Then, please run f.est() and f.plot.simrsl() functions. If you edit f.est() function, from f.est <- function(beta=1, n=40000) to f.est <- function(beta, n), you can use beta and n as arguments when you run the function.
