# Supplementary material for: A neuronal prospect theory model in the brain reward circuitry
Source: Nat Commun. 2022 Oct 4;13:5855. doi: 10.1038/s41467-022-33579-0 (PMC9532451; doi:10.1038/s41467-022-33579-0)
Supplement: Supplementary file 7 — Reporting Summary [file 41467_2022_33579_MOESM7_ESM.pdf]

nature portfolio

Corresponding author(s): Hiroshi Yamada

Last updated by author(s): Aug 21, 2022

Reporting Summary

Nature Portfolio wishes to improve the reproducibility of the work that we publish. This form provides structure for consistency and transparency in reporting. For further information on Nature Portfolio policies, see our Editorial Policies and the Editorial Policy Checklist.

Statistics

For all statistical analyses, confirm that the following items are present in the figure legend, table legend, main text, or Methods section.

n/a	Confirmed	
☐	☒	The exact sample size (n) for each experimental group/condition, given as a discrete number and unit of measurement
☐	☒	A statement on whether measurements were taken from distinct samples or whether the same sample was measured repeatedly
☐	☒	The statistical test(s) used AND whether they are one- or two-sided<br>*Only common tests should be described solely by name; describe more complex techniques in the Methods section.*
☐	☒	A description of all covariates tested
☐	☒	A description of any assumptions or corrections, such as tests of normality and adjustment for multiple comparisons
☐	☒	A full description of the statistical parameters including central tendency (e.g. means) or other basic estimates (e.g. regression coefficient) AND variation (e.g. standard deviation) or associated estimates of uncertainty (e.g. confidence intervals)
☐	☒	For null hypothesis testing, the test statistic (e.g. F, t, r) with confidence intervals, effect sizes, degrees of freedom and P value noted<br>*Give P values as exact values whenever suitable.*
☒	☐	For Bayesian analysis, information on the choice of priors and Markov chain Monte Carlo settings
☒	☐	For hierarchical and complex designs, identification of the appropriate level for tests and full reporting of outcomes
☒	☐	Estimates of effect sizes (e.g. Cohen's d, Pearson's r), indicating how they were calculated

Our web collection on statistics for biologists contains articles on many of the points above.

Software and code

Policy information about availability of computer code

Data collection	We used Matlab R2015b with Psychtoolbox 3.0 for behavioral task control. Open developer software 2.16, OpenEx 2.16, and OpenSorter 2.16 were used in TDT system for data collection.
Data analysis	We have already uploaded all analysis code files used in R. All data were analyzed with Matlab R2015b for preprocessing and R 3.6.2 for statistical test. Simulations were made using R 3.6.2.

For manuscripts utilizing custom algorithms or software that are central to the research but not yet described in published literature, software must be made available to editors and reviewers. We strongly encourage code deposition in a community repository (e.g. GitHub). See the Nature Portfolio guidelines for submitting code & software for further information.

Data

Policy information about availability of data

All manuscripts must include a data availability statement. This statement should provide the following information, where applicable:

- Accession codes, unique identifiers, or web links for publicly available datasets
- A description of any restrictions on data availability
- For clinical datasets or third party data, please ensure that the statement adheres to our policy

Data and Code are provided in the Supplementary Information/Source Data file. Please see the data and code availability statement in the manuscript.

Human research participants

Policy information about studies involving human research participants and Sex and Gender in Research.

Reporting on sex and gender	N/A
Population characteristics	N/A
Recruitment	N/A
Ethics oversight	N/A

Note that full information on the approval of the study protocol must also be provided in the manuscript.

Field-specific reporting

Please select the one below that is the best fit for your research. If you are not sure, read the appropriate sections before making your selection.

☒ Life sciences ☐ Behavioural & social sciences ☐ Ecological, evolutionary & environmental sciences

For a reference copy of the document with all sections, see nature.com/documents/nr-reporting-summary-flat.pdf

Life sciences study design

All studies must disclose on these points even when the disclosure is negative.

Sample size	We describe the sample size for each neuron, number of neurons in each brain region, and these values in each monkey. The sample size was defined with our previous study. We denote this in the method section as follows. The sample sizes required to detect effect sizes (number of recorded neurons, number of recorded trials in a single neuron, and number of monkeys) were estimated in reference to previous studies, referecne No. 44, 55, 56. Neural activity was recorded during the 100–120 trials of the single-cue task. The sample sizes required to detect effect sizes (number of recorded neurons, number of recorded trials in a single neuron, and number of monkeys) were estimated in reference to previous studies No. 44, 55, 56. Neural activity was recorded during the 100–120 trials of the single-cue task.
Data exclusions	We did not exclude the data.
Replication	We confirmed our results by compering two monkeys. In each monkey, we performed experiment in reference to number of neurons recorded in each brain regions (50-100 neurons), number of trials recorded in each neuron (100–120 trials), and all related statistical significance for these measures ($P < 0.05$).
Randomization	This is not relevant for neural recordings because all neurons were recorded if the recording condition satisfied the following criterion. We denoted that the criterion of neural recordings in method section as follows. The activity of all the single neurons was sampled when the activity of an isolated neuron demonstrated a good signal-to-noise ratio (> 2.5).
Blinding	Blinding was not performed because it is not possible for neural recording.

Reporting for specific materials, systems and methods

We require information from authors about some types of materials, experimental systems and methods used in many studies. Here, indicate whether each material, system or method listed is relevant to your study. If you are not sure if a list item applies to your research, read the appropriate section before selecting a response.

Materials & experimental systems

n/a	Involved in the study	
☒	☐	Antibodies
☒	☐	Eukaryotic cell lines
☒	☐	Palaeontology and archaeology
☐	☒	Animals and other organisms
☒	☐	Clinical data
☒	☐	Dual use research of concern

Methods

n/a	Involved in the study	
☒	☐	ChIP-seq
☒	☐	Flow cytometry
☒	☐	MRI-based neuroimaging

March 2021

Animals and other research organisms

Policy information about studies involving animals; ARRIVE guidelines recommended for reporting animal research, and Sex and Gender in Research

Laboratory animals	We denoted this information in method section. Two rhesus monkeys (Macaca mulatta, SUN, 7.1 kg, male, during 4-8 years old; Macaca fuscata, FU, 6.7 kg, female, during 4-7 years old).
Wild animals	Home caged monkeys were used.
Reporting on sex	Two rhesus monkeys (Macaca mulatta, SUN, 7.1 kg, male, during 4-8 years old; Macaca fuscata, FU, 6.7 kg, female, during 4-7 years old).
Field-collected samples	No field collected data was used in this study.
Ethics oversight	We denoted this information in the method section as follows. All experimental procedures were approved by the Animal Care and Use Committee of the University of Tsukuba (Protocol No. H30.336) and performed in compliance with the US Public Health Service's Guide for the Care and Use of Laboratory Animals.

Note that full information on the approval of the study protocol must also be provided in the manuscript.
